# Genetic predictors of cardiovascular morbidity in Bardet–Biedl syndrome

**DOI:** 10.1111/cge.12373

**Published:** 2014-04-08

**Authors:** E Forsythe, K Sparks, BE Hoskins, E Bagkeris, BM McGowan, PV Carroll, MSB Huda, S Mujahid, C Peters, T Barrett, S Mohammed, PL Beales

**Affiliations:** a Molecular Medicine Unit, UCL Institute of Child Health, University College LondonLondon, UK; b National Bardet-Biedl Syndrome Service, Great Ormond Street HospitalLondon, UK; c MRC Centre of Epidemiology for Child Health, UCL Institute of Child Health, University College LondonLondon, UK; d Department of Endocrinology, Guy's HospitalLondon, UK; e Department of Endocrinology, St Bartholomew and the Royal London HospitalsLondon, UK; f Department of Endocrinology, Great Ormond Street HospitalLondon, UK; g Diabetes Unit, Birmingham Children's HospitalBirmingham, UK; h Department of Clinical Genetics, Guy's HospitalLondon, UK

**Keywords:** Bardet–Biedl syndrome, cardiovascular morbidity, genotype–phenotype correlation, mutation type

## Abstract

Bardet–Biedl syndrome is a rare ciliopathy characterized by retinal dystrophy, obesity, intellectual disability, polydactyly, hypogonadism and renal impairment. Patients are at high risk of cardiovascular disease. Mutations in *BBS1* and *BBS10* account for more than half of those with molecular confirmation of the diagnosis. To elucidate genotype–phenotype correlations with respect to cardiovascular risk indicators 50 patients with mutations in *BBS1* were compared with 19 patients harbouring *BBS10* mutations. All patients had truncating, missense or compound missense/truncating mutations. The effect of genotype and mutation type was analysed. C-reactive protein was higher in those with mutations in *BBS10* and homozygous truncating mutations (p = 0.013 and p = 0.002, respectively). Patients with mutations in *BBS10* had higher levels of C peptide than those with mutations in *BBS1* (p = 0.043). Triglyceride levels were significantly elevated in patients with homozygous truncating mutations (p = 0.048). Gamma glutamyl transferase was higher in patients with homozygous truncating mutations (p = 0.007) and heterozygous missense and truncating mutations (p = 0.002) than those with homozygous missense mutations. The results are compared with clinical cardiovascular risk factors. Patients with missense mutations in *BBS1* have lower biochemical cardiovascular disease markers compared with patients with *BBS10* and other *BBS1* mutations. This could contribute to stratification of the clinical service.

Bardet–Biedl syndrome (BBS) is a pleiotropic autosomal recessive ciliopathy characterized by retinal dystrophy, post-axial polydactyly, obesity, learning difficulties, hypogonadism and renal dysfunction (1, 2). There is a high prevalence of cardiovascular, endocrine and renal disorders among patients with BBS (3). In order to optimize the clinical management, it is imperative to identify patients who are most at risk of disease-associated morbidity and mortality.

Since the clinical criteria for a diagnosis were proposed, 19 genes (*BBS1–BBS19*) have been discovered (4, 5). BBS genes code for proteins that localize to the basal body of the cilium. Mutations lead to defective cilia accounting in part for the variable effects observed in BBS. A clinical diagnosis can be confirmed by sequencing the known disease-causing genes in 80% of patients (authors' own unpublished data).

The majority of pathogenic mutations are found in *BBS1* and *BBS10* accounting for 23.2% and 20%, respectively in populations of northern European descent (4). The commonest mutation is M390R found in 82.5% of a cohort of British patients with *BBS1* mutations (4). The frameshift mutation C91LfsX5 is prevalent in patient populations with *BBS10* mutations.

Variable phenotypic expressivity is a hallmark of BBS, however, even among patients with the same genotype, interfamilial and intrafamilial phenotypic variability is common. Mutations in other BBS genes may modify the phenotype, accounting for this variability (6). Several studies have attempted to identify a genotype–phenotype correlation in BBS (7–10). These have primarily focused on physical features and have been limited by small sample sizes or participants from the same kindred.

This is the first study to explore the correlation between genotype, mutation type and morbidity in BBS. We examine indicators of cardiovascular, metabolic and renal morbidity in a large cohort of patients with BBS and compare the two most commonly mutated genes: *BBS1* and *BBS10*.

## Methods

### Patients

Two hundred and thirty nine patients attending the national Bardet-Biedl Syndrome clinics in London and Birmingham were assessed for height, weight, blood pressure, BBS mutation analysis, full blood count, renal function, liver function, inflammatory markers, endocrine and lipid profile. Information on cardiovascular risk factors was collected retrospectively from patient notes. The following clinical parameters were ascertained: (i) hypertension (defined as a blood pressure over 140/90 or normotensive requiring antihypertensive medication), (ii) hypercholesterolaemia requiring hypolipidaemic agents, (iii) diabetes mellitus requiring hypoglycaemic medication, (iv) structural renal abnormalities and/or dialysis or renal transplant, and (v) structural cardiac abnormalities. Patients were predominantly of Caucasian origin. Referrals were made primarily via the British national patient support group and clinical geneticists in the United Kingdom.

### Mutation screening

Eighty four of the 239 patients had two known pathogenic mutations in BBS genes. Of these, 73 harboured two mutations in *BBS1* or *BBS10*. Mutation analysis was primarily undertaken through targeted sequencing of the four most common mutations: M390R in *BBS1*, and Y24X and R275X in *BBS2* and C91LfsX5 in *BBS10*. Where only one mutation was found, full sequencing of the relevant gene was performed to identify a second mutation.

### Statistical analysis

Analysis was targeted to *BBS1* and *BBS10* patients with truncating and/or missense mutations to allow for adequate sample sizes.

We applied a two pronged approach to statistical analysis. Mann–Whitney *U* test, Kruskal–Wallis test and analysis of variance (anova) were performed as appropriate to identify associations between genes (*BBS1 vs BBS10*) and mutation types (homozygous truncating mutations, heterozygous missense and truncating mutations or homozygous missense mutations). Multivariable linear regression analysis was applied to variables which were statistically significant on univariable analysis and/or known indicators of metabolic, renal and cardiovascular disease. Statistical analyses were carried out using spss version 21.0 (SPSS Inc., Chicago IL).

## Results

### Distribution of patients

**Fig 1:**
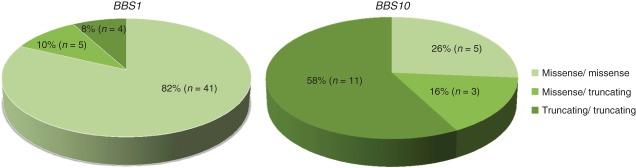
Distribution of mutation type in patients with *BBS1* and *BBS10* included in the analysis.

Fifty two patients harboured two mutations in *BBS1* and 21 harboured two mutations in *BBS10*. This included nine pairs and two sets of three siblings. DNA results were classified according to gene and mutation. Mutation type was further classified according to the predicted severity. Most patients had either homozygous missense mutations, homozygous truncating mutations or a heterozygous missense and truncating mutations. Two patients with *BBS1* mutations and two patients with *BBS10* mutations harboured splice site mutations and were excluded from the statistical analysis. Figure 1 demonstrates the distribution of mutation types in the *BBS1* and *BBS10* genotypes analysed in this study, illustrating the higher proportions of missense mutations in *BBS1* and truncating mutations in *BBS10*.

**Fig 2:**
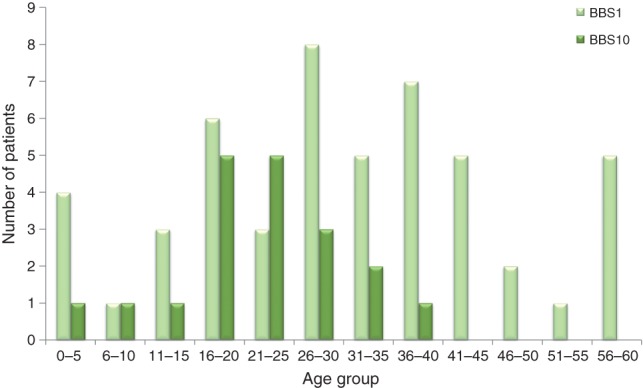
Age distribution of patients with mutations in *BBS1* and *BBS10* included in the analysis.

Of the remaining 69 patients seen in the clinic with a mutation in *BBS1* or *BBS10* the mean age was 28.25 (SD: 14.41, range: 0–59). The mean (SD) age of patients with a *BBS1* mutation was 30.5 (15.6) years. In contrast the mean age (SD) of patients with a mutation in *BBS10* was 22.32 (8.95) years (p = 0.034). Figure 2 illustrates the age distribution of patients with *BBS1* and *BBS10* included in this study. Twenty four (48%) patients with a mutation in *BBS1* were female and 26 (52%) were male. Twelve (63.2%) patients with mutations in *BBS10* were female and seven (36.8%) were male.

### Clinical parameters

Genotype–phenotype associations were tested for all clinical variables. Univariable analysis comparing patients with *BBS1 vs BBS10* demonstrated a statistically significant difference in age, height, C-reactive protein (CRP), c-peptide, triglycerides, potassium, and albumin–creatinine ratio (Table 1). Comparison of mutation types revealed a statistically significant difference in high density lipoprotein (HDL) cholesterol and gamma glutamyl transferase (GGT) (Table 2).

**Table 1:** Genotype–phenotype correlation: univariable comparison of statistically significant parameters. Patients with *BBS1 vs BBS10*. Only statistically significant results are included. The full analysis is available in Tables S1, Supporting information

	BBS 1		BBS 10		
	Mean	SD	Mean	SD	p-Value^a^
Anthropomorphic measurements					
Age at clinic	30.5	−15.48	22.32	−8.95	0.034
Height (cm)	163.92	−28.17	162.93	−9.31	0.031
Inflammatory markers					
CRP (mg/l)	5.69	−2.74	9.53	−7.12	0.04
Endocrine profile					
C peptide (ng/ml)	1295.88	−740.13	2333.3	−1501.27	0.014
Lipid profile					
Triglycerides (mmol/l)	1.5	−0.73	1.98	−0.94	0.049
Renal profile					
Potassium (mmol/l)	4.16	−0.48	4.38	−0.42	0.015
Albumin/creatinine ratio	7.1	−22.39	5.3	−11.23	0.032

ANOVA, analysis of variance; BBS, Bardet–Biedl syndrome, CRP, C-reactive protein.

a p-Value obtained from anova test or Mann–Whitney *U* test.

**Table 2:** Mutation type-phenotype comparison: univariable comparison of statistically significant parameters. Homozygous missense; heterozygous truncating and missense and homozygous truncating mutations. Only statistically significant results are included. The full analysis is available in Table S2

	Missense/missense		Missense/truncating		Truncating/truncating		
	Mean	SD	Mean	SD	Mean	SD	p-Value^a^
Lipid profile							
HDL cholesterol (mmol/l)	1.28	−0.25	1.09	−0.12	1.1	−0.23	0.022
Liver profile							
Gamma glutamyl transferase (U/l)	29.21	−16.22	70.33	−10.02	62.75	−40.01	0.027

HDL, high-density lipoprotein.

a p-value obtained from anova test or Kruskal–Wallis test.

We applied multivariable analysis to selected variables based on association with cardiovascular risk and controlled for confounding factors. Statistically significant results are displayed in Tables 3 and 4 and discussed here.

**Table 3:** Genotype–phenotype comparison: multivariable comparison of selected parameters found to be statistically significant. *BBS1 vs BBS10*. Only statistically significant results are included in this table. The full analysis is available in Table S3

	β estimate	95.0% CI	p-Value^a^
CRP (mg/l)			
Genotype			
BBS1	Reference	—	—
BBS10	**4.08**	**(0.90, 7.25)**	**0.013**
Age	0.06	(−0.05, 0.18)	0.295
BMI	0.1	(−0.08, 0.29)	0.266
C peptide (ng/ml)			
Genotype			
BBS1	Reference	—	—
BBS10	**942.94**	**(32.26, 1853.61)**	**0.043**
BMI	−4.86	(−67.84, 58.12)	0.876
Blood glucose	92.08	(−71.24, 255.41)	0.258

BBS, Bardet–Biedl syndrome; BMI, body mass index; CI, confidence interval; CRP, C-reactive protein. Significant values are highlighted in bold.

a p-value obtained from linear regression model.

**Table 4:** Mutation type-phenotype comparison: multivariable comparison of selected parameters found to be statistically significant. Homozygous missense; heterozygous truncating and missense and homozygous truncating. Only statistically significant results are included in this table. The full analysis is available as Table S4

	β estimate	95.0% CI	p-Value^a^
CRP (mg/l)			
Mutation type			
Missense/missense	Reference	—	—
Missense/truncating	−0.65	(−4.42, 3.12)	0.729
Truncating/truncating	**5.33**	**(1.99, 8.68)**	**0.002**
Age	0.06	(−0.05, 0.17)	0.272
BMI	0.14	(−0.03, 0.31)	0.11
Triglycerides (mmol/l)			
Mutation type			
Missense/missense	Reference	—	—
Missense/truncating	0	(−0.67, 0.67)	0.996
Truncating/truncating	**0.56**	**(0.01, 1.11)**	**0.048**
Gender			
Female	Reference	—	—
Male	**0.52**	**(0.07, 0.98)**	**0.026**
BMI	**0.03**	**(0.00, 0.06)**	**0.05**
Age	−0.01	(−0.03, 0.01)	0.452
Gamma glutamyl transferase (U/l)			
Mutation type			
Missense/missense	Reference	—	—
Missense/truncating	**44.22**	**(17.90, 70.54)**	**0.002**
Truncating/truncating	**29.32**	**(8.72, 49.91)**	**0.007**
Gender			
Female	Reference	—	—
Male	**17.94**	**(4.12, 31.76)**	**0.013**
BMI	**1.05**	**(0.15,1.95)**	**0.025**
Age	0.24	(−0.28, 0.76)	0.349

Significant values are highlighted in bold.

a p-Value obtained from linear regression model.

#### Inflammatory markers

A statistically significant difference in CRP is observed. Patients with a mutation in *BBS10* or homozygous truncating mutations have a significantly higher CRP (p = 0.013 and p = 0.002, respectively). Analysis of white cell count and other blood count parameters did not reach statistical significance.

#### C peptide

C peptide levels were significantly higher in patients with *BBS10* compared with *BBS1* mutations (p = 0.043).

#### Lipid profile

Triglycerides levels were significantly higher in patients with homozygous truncating mutations (p = 0.048) than those with other mutation types.

#### Liver function

Multivariable analysis demonstrated significantly higher GGT in patients with homozygous truncating or heterozygous missense and truncating mutations than those with homozygous missense mutations (p = 0.007 and p = 0.002, respectively).

### Clinical cardiovascular risk factors

**Fig 3:**
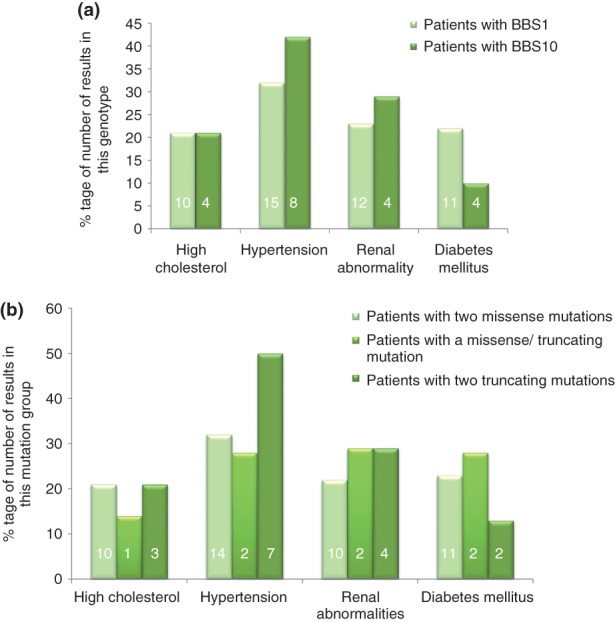
Prevalence of cardiovascular risk factors by genotype (a) and mutation type (b). The prevalence is illustrated as a percentage to compensate for disproportionate population sizes. Absolute numbers are given in each column.

Sixty seven patients had a full lipid profile, of whom 14 had hypercholesterolaemia. Hypertension was identified in 23 of 65 patients. Fifteen of 69 patients had a diagnosis of diabetes mellitus. Five of 69 patients had the results of an echocardiogram documented. Of these, one had an innocent murmur, two had a ventricular septal defect, one had an atrioventricular septal defect and one patient had aortic valve stenosis. All of these patients had two missense mutations in *BBS1*. Twenty nine patients had renal ultrasounds. Sixteen of these had abnormal results ranging from benign structural malformations to sonographic evidence of chronic renal failure. Two patients had renal transplants and one was on dialysis, all of whom had two truncating mutations in *BBS10*. Figure 3a,b illustrate the prevalence of the clinical cardiovascular risk factors. Cardiac abnormalities are not included in these diagrams as only five patients had documented echocardiograms and we therefore perceive the results not be representative of the group as a whole.

## Discussion

To our knowledge this is the only study comparing cardiovascular risk factors for patients with *BBS1* and *BBS10* – the two most commonly mutated genes in patients from Europe and North America. It is the first published study to compare the BBS phenotype according to mutation type. Several studies have suggested that there is little evidence of a genotype–phenotype correlation in BBS and proposed that this may be because BBS proteins contribute to a common molecular pathway (4). However, there is an emerging evidence of some genotype–phenotype correlations (7, 10). Feuillian et al. (10) reported that patients with *BBS1* had lower insulin resistance compared with patients with *BBS10*. Observations from the British nationally commissioned clinic suggest that patients with two mutations in *BBS10* are often more severely affected than those with *BBS1* although there is considerable variation.

CRP increases with obesity and inflammation. Chronically raised CRP indicates a higher risk of cardiovascular morbidity (11). This study shows that patients with *BBS10* genotypes and/or two truncating mutations have a significantly worse CRP value. As CRP is a physiological marker of inflammation and infection, it is notable that there was no statistically significant difference in white cell count or weight between patients with different genes or mutation types. This suggests that patients with missense mutations in *BBS1* may be at lower risk of cardiovascular disease than patients with *BBS10* or other mutations in *BBS1*.

C peptide is used as a marker of insulin resistance but has in recent years been recognized as an independent bioactive peptide exerting effects on microvascular function, correlating with macrovascular complications and cardiovascular death (12). Our results demonstrate that patients with mutations in *BBS10* have significantly higher levels of C-peptide indicating insulin resistance, supporting the suggestion that they are at higher risk of cardiovascular disease than patients with mutations in *BBS1*.

Raised triglycerides are associated with an increased risk of cardiovascular disease (13). Our results demonstrate that patients with homozygous truncating mutations are more likely to have raised triglycerides than patients with other mutation types.

We demonstrated a statistically significant increase in GGT in patients with homozygous truncating mutations and heterozygous truncating and missense mutations compared with patients with homozygous missense mutations. Although used as a marker of chronic liver disease, GGT correlates with cardiovascular diseases and is an independent marker of cardiovascular risk (14) making it a potentially powerful tool in the risk stratification of patients with BBS.

The prevalence of clinical cardiovascular risk factors according to genotype and mutation type do not reveal a clear pattern. This may be because the patients in this study are young (mean age 28.25) and the natural progression of the disease has not yet unfolded, or because the patients with *BBS1* mutations are significantly older than the patients with mutations in *BBS10*. Alternatively, it may reflect a true lack of genotype–phenotype correlation.

Although analysis of renal parameters did not reveal any statistically significant differences, it is noteworthy that the patient on dialysis and both patients who had received renal transplants had two truncating mutations in *BBS10*. This is in keeping with previous studies suggesting that the renal phenotype may be more severe in patients with mutations in *BBS10* (3).

The high prevalence of hypertension (Figure 3a,b) among patients with mutations in *BBS10* and two truncating mutations (42% and 50%) respectively is striking considering only one person in these groups is older than 31. This may represent the early development of a severe cardiovascular phenotype or a statistical error due to sample sizes (19 and 14, respectively).

## Conclusion

Cardiovascular disease is a major cause of death and morbidity, and significant resources are allocated to primary prevention in the general population with the aim of reducing the overall disease burden. The same principles should apply to special groups within the population such as patients with BBS where there is an opportunity to practice personalized medicine as the genotype of many patients is already known. This study indicates that patients with missense mutations in *BBS1* may be at lower risk of cardiovascular disease than patients with homozygous truncating mutations and mutations in *BBS10*. In practice most patients with *BBS10* mutations harbour the common homozygous truncating mutation and it is possible that the resulting truncated protein product or a hypomorphic effect of the common missense mutation M390R in *BBS1*, rather than the affected gene, determines the phenotypic effect. Larger studies could clarify this, and longitudinal research will determine the clinical effect these risk factors have on cardiovascular morbidity.

## Supporting Information

The following Supporting information is available for this article:

Table S1. Genotype–phenotype correlation: univariable comparison of clinical and laboratory parameters. Patients with *BBS1 vs* *BBS10*. Statistically significant results are highlighted in bold.

Table S2. Mutation type-phenotype comparison: univariable comparison of clinical and laboratory parameters. Homozygous missense, heterozygous truncating and missense and homozygous truncating mutations. Statistically significant results are highlighted in bold.

Table S3. Genotype–phenotype comparison: multivariable comparison of selected parameters associated with cardiovascular disease. *BBS1* *vs* *BBS10*. Statistically significant results are highlighted in bold.

Table S4. Mutation type-phenotype comparison: multivariable comparison of selected parameters associated with cardiovascular disease. Homozygous missense, heterozygous truncating and missense and homozygous truncating. Statistically significant results are highlighted in bold.
