# Supplementary material for: Genetic predictors of cardiovascular morbidity in Bardet–Biedl syndrome
Source: Clin Genet. 2014 Apr 8;87(4):343–9. doi: 10.1111/cge.12373 (PMC4402025; doi:10.1111/cge.12373)
Supplement: Supplementary file 1 — Table S1. Genotype–phenotype correlation: univariable comparison of clinical and laboratory parameters. Patients with BBS1 vs BBS10. Statistically significant results are highlighted in bold. [file cge0087-0343-sd1.doc]

Table 1

|  | **BBS 1** | | **BBS 10** | |  |
| --- | --- | --- | --- | --- | --- |
|  | **Mean** | **(SD)** | **Mean** | **(SD)** | **p-value*** |
| **Anthropomorphic measurements** | | | | | |
| Age at Clinic | **30.5** | **-15.48** | **22.32** | **-8.95** | **0.034** |
| Systolic blood pressure (mmHg) | 122.89 | -13.87 | 117.69 | -17.42 | 0.411 |
| Diastolic blood pressure (mmHg) | 77.47 | -11.08 | 78.27 | -9.4 | 0.676 |
| Height (cm) | **163.92** | **-28.17** | **162.93** | **-9.31** | **0.031** |
| Weight (kg) | 93 | -35.64 | 94.22 | -31.48 | 0.934 |
| BMI | 32.69 | -8.08 | 36.17 | -8.16 | 0.125 |
| **Inflammatory markers** | | | | | |
| White cell count (10^9^L) | 7.23 | -1.85 | 8.28 | -2.26 | 0.074 |
| CRP (mg/L) | **5.69** | **-2.74** | **9.53** | **-7.12** | **0.04** |
| Platelets (10^9^L) | 241.82 | -65.28 | 233.2 | -51.04 | 0.649 |
| **Endocrine profile** | | | | | |
| Cortisol (nmol/L) | 382.22 | -154.94 | 302.69 | -128.17 | 0.094 |
| T3 (µg/dL) | 5.18 | -0.51 | 4.65 | -1.06 | 0.507 |
| C peptide (ng/ml) | **1295.88** | **-740.13** | **2333.3** | **-1501.27** | **0.014** |
| Insulin (mmol/L) | 135.19 | -108.32 | 308.22 | -462.62 | 0.082 |
| HbA1c (%Hb) | 5.75 | -0.67 | 5.86 | -0.98 | 0.935 |
| Blood glucose (mmol/L) | 5.34 | -1.81 | 5.71 | -2.39 | 0.563 |
| **Lipid Profile** | | | | | |
| Cholesterol (mmol/L) | 4.6 | -0.79 | 4.65 | -0.84 | 0.827 |
| Triglycerides (mmol/L) | **1.5** | **-0.73** | **1.98** | **-0.94** | **0.049** |
| HDL cholesterol (mmol/L) | 1.24 | -0.23 | 1.14 | -0.27 | 0.163 |
| LDL cholesterol (mmol/L) | 2.69 | -0.81 | 2.62 | -0.71 | 0.722 |
| **Renal profile** | | | | | |
| Sodium (mmol/L) | 141.98 | -2.97 | 141.83 | -2.85 | 0.863 |
| Potassium (mmol/L) | **4.16** | **-0.48** | **4.38** | **-0.42** | **0.015** |
| Estimated GFR | 88.48 | -24.71 | 80.38 | -33.49 | 0.371 |
| Calcium (mmol/L) | 2.2 | -0.23 | 2.17 | -0.28 | 1 |
| Magnesium (mmol/L) | 0.9 | -0.08 | 0.88 | -0.13 | 0.232 |
| Phosphate (mmol/L) | 1.15 | -0.31 | 1.35 | -0.63 | 0.213 |
| Albumin/Creatinine ratio | **7.1** | **-22.39** | **5.3** | **-11.23** | **0.032** |
| Creatinine (µmol/L) | 77.7442 | 30.501 | 103.56 | 108.318 | 1 |
| Urea (mmol/L) | 5.6913 | 3.1569 | 6.3125 | 3.11009 | 0.278 |
| **Liver profile** | | | | | |
| Albumin (g/L) | 47.12 | -3.3 | 45.83 | -2.55 | 0.146 |
| Total bilirubin (µmol/L) | 8.86 | -6.7 | 8.94 | -5.59 | 0.842 |
| Alkaline Transaminase (IU/L) | 36.59 | -28.76 | 34.67 | -28.36 | 0.698 |
| Gamma Glutamyl Transferase (U/L) | 34.19 | -20.63 | 54.8 | -38.94 | 0.375 |

*p-value obtained from ANOVA test or Mann Whitney U test
