# Supplementary material for: Genetic predictors of cardiovascular morbidity in Bardet–Biedl syndrome
Source: Clin Genet. 2014 Apr 8;87(4):343–9. doi: 10.1111/cge.12373 (PMC4402025; doi:10.1111/cge.12373)
Supplement: Supplementary file 2 — Table S2. Mutation type-phenotype comparison: univariable comparison of clinical and laboratory parameters. Homozygous missense, heterozygous truncating and missense and homozygous truncating mutations. Statistically significant results are highlighted in bold. [file cge0087-0343-sd2.doc]

**Table 2**

|  | **Missense/ missense** | | **Missense/ nonsense** | | **Nonsense/ nonsense** | |  |
| --- | --- | --- | --- | --- | --- | --- | --- |
|  | **Mean** | **(SD)** | **Mean** | **(SD)** | **Mean** | **(SD)** | **p-value*** |
| **Anthropomorphic measurements** | | | | | | | |
| Age at Clinic | 30.52 | -15.6 | 28.25 | -10.99 | 21.27 | -9.79 | 0.096 |
| Systolic blood pressure (mmHg) | 123.59 | -13.46 | 116.5 | -13.38 | 118.64 | -19.05 | 0.211 |
| Diastolic blood pressure (mmHg) | 78.59 | -11.2 | 73.5 | -6.57 | 77.62 | -10.87 | 0.493 |
| Height (cm) | 163.12 | -27.92 | 167.43 | -16.74 | 163.12 | -9.67 | 0.278 |
| Weight (kg) | 95.33 | -33.76 | 99.2 | -36.02 | 84.56 | -35.62 | 0.289 |
| BMI | 33.84 | -7.88 | 34.29 | -9.17 | 33.26 | -9.21 | 0.959 |
| **Inflammatory markers** | | | | | | | |
| White cell count (10^9^L) | 7.22 | -1.72 | 8.41 | -2.47 | 7.87 | -2.41 | 0.269 |
| CRP (mg/L) | 5.94 | -2.9 | 5 | -1.07 | 10.25 | -7.83 | 0.309 |
| Platelets (10^9^L) | 235.74 | -65.97 | 269.33 | -71.98 | 235.08 | -40.15 | 0.453 |
| **Endocrine profile** | | | | | | | |
| Cortisol (nmol/L) | 371.03 | -164.4 | 325.43 | -82.59 | 344.5 | -149.39 | 0.87 |
| T3 (µg/dL) | 5.23 | -0.49 | 4.7 | -0.57 | 4.65 | -1.06 | 0.455 |
| C peptide (ng/ml) | 1436.8 | -849.7 | 1257.8 | -402.61 | 2052.7 | -1602.64 | 0.479 |
| Insulin (mmol/L) | 151.79 | -110.4 | 121.4 | -95.21 | 291.03 | -491.4 | 0.848 |
| HbA1c (%Hb) | 5.82 | -0.69 | 5.63 | -0.83 | 5.74 | -0.96 | 0.54 |
| Blood glucose | 5.36 | -1.93 | 5.86 | -2.59 | 5.47 | -1.96 | 0.944 |
| **Lipid profile** | | | | | | | |
| Cholesterol (mmol/L) | 4.65 | -0.75 | 4.38 | -0.73 | 4.65 | -0.98 | 0.669 |
| Triglycerides (mmol/L) | 1.5 | -0.71 | 1.63 | -1.13 | 1.99 | -0.86 | 0.158 |
| HDL cholesterol (mmol/L) | **1.28** | **-0.25** | **1.09** | **-0.12** | **1.1** | **-0.23** | **0.022** |
| LDL cholesterol (mmol/L) | 2.7 | -0.79 | 2.55 | -0.73 | 2.65 | -0.8 | 0.881 |
| **Renal profile** | | | | | | | |
| Na (mmol/L) | 141.42 | -2.93 | 142.38 | -2.5 | 143 | -2.93 | 0.187 |
| K (mmol/L) | 4.17 | -0.49 | 4.15 | -0.4 | 4.39 | -0.45 | 0.101 |
| eGFR | 88 | -23.08 | 85.71 | -38.21 | 81.91 | -31.9 | 0.827 |
| Calcium (mmol/L) | 2.19 | -0.25 | 2.1 | -0.35 | 2.26 | -0.06 | 0.388 |
| Magnesium (mmol/L) | 0.9 | -0.08 | 0.88 | -0.16 | 0.89 | -0.12 | 0.652 |
| Phosphate (mmol/L) | 1.17 | -0.31 | 1.11 | -0.26 | 1.35 | -0.68 | 0.743 |
| Alb/Cr ratio | 3.83 | -7.99 | 23.48 | -51.33 | 4.57 | -10.85 | 0.421 |
| Creatinine (µmol/L) | 76.32 | 24.19 | 94.38 | 62.11 | 103.47 | 116.39 | 0.925 |
| Urea (mmol/L) | 5.43 | 2.67 | 8.43 | 6.01 | 5.62 | 1.27 | 0.486 |
| **Liver profile** | | | | | | | |
| Albumin (g/L) | 47 | -3.27 | 47.5 | -2.73 | 45.67 | -2.89 | 0.293 |
| Total bilirubin (µmol/L) | 8.53 | -6.53 | 10.38 | -7.42 | 9 | -5.53 | 0.796 |
| Alkaline Transaminase (IU/L) | 32.03 | -19.11 | 47.88 | -38.06 | 40.13 | -40.76 | 0.367 |
| Gamma Glutamyl Transferase (U/L) | **29.21** | **-16.22** | **70.33** | **-10.02** | **62.75** | **-40.01** | **0.027** |
| Alb/Cr ratio | 3.83 | -7.99 | 23.48 | -51.33 | 4.57 | -10.85 | 0.421 |

*p-value obtained from ANOVA test or Kruskal-Wallis test
