# Supplementary material for: Genetic predictors of cardiovascular morbidity in Bardet–Biedl syndrome
Source: Clin Genet. 2014 Apr 8;87(4):343–9. doi: 10.1111/cge.12373 (PMC4402025; doi:10.1111/cge.12373)
Supplement: Supplementary file 3 — Table S3. Genotype–phenotype comparison: multivariable comparison of selected parameters associated with cardiovascular disease. BBS1 vs BBS10. Statistically significant results are highlighted in bold. [file cge0087-0343-sd3.doc]

Table 3

|  | **β Estimate** | **95.0% CI** | **p-value*** |
| --- | --- | --- | --- |
| **Systolic blood pressure (mmHg)** | | | |
| Genotype |  |  |  |
| BBS1 | *Reference* | *-* | *-* |
| BBS10 | -2.97 | (-11.74, 5.79) | 0.499 |
| Age | 0.16 | (-0.18, 0.49) | 0.351 |
| Height | **20.90** | **(1.76, 40.04)** | **0.033** |
| Gender |  |  |  |
| Female | *Reference* | *-* | *-* |
| Male | 3.47 | (-3.95, 10.90) | 0.352 |
| BMI | 0.398 | (-0.14, 0.94) | 0.145 |
| **Diastolic blood pressure (mmHg)** | | | |
| Genotype |  |  |  |
| BBS1 | *Reference* | *-* | *-* |
| BBS10 | 3.19 | (-4.05, 10.42) | 0.380 |
| Age | **0.30** | **(0.03, 0.57)** | **0.032** |
| Height | -1.02 | (-20.46, 18.43) | 0.917 |
| Gender |  |  |  |
| Female | *Reference* | *-* | *-* |
| Male | 2.04 | (-4.11, 8.18) | 0.508 |
| BMI | -0.01 | (-0.44, 0.44) | 0.995 |
| **White cell count (10^9^L)** | | | |
| Genotype |  |  |  |
| BBS1 | *Reference* | *-* | *-* |
| BBS10 | 1.01 | (-0.19, 2.22) | 0.098 |
| Age | -0.01 | (-0.05, 0.03) | 0.789 |
| **CRP (mg/L)** | | | |
| Genotype |  |  |  |
| BBS1 | *Reference* | *-* | *-* |
| BBS10 | **4.08** | **(0.90, 7.25)** | **0.013** |
| Age | 0.06 | (-0.05, 0.18) | 0.295 |
| BMI | 0.1 | (-0.08, 0.29) | 0.266 |
| **C peptide (ng/ml)** | | | |
| Genotype |  |  |  |
| BBS1 | *Reference* | *-* | *-* |
| BBS10 | **942.94** | **(32.26, 1853.61)** | **0.043** |
| BMI | -4.86 | (-67.84, 58.12) | 0.876 |
| Blood glucose | 92.08 | (-71.24, 255.41) | 0.258 |
| **Cholesterol (mmol/L)** | | | |
| Genotype |  |  |  |
| BBS1 | *Reference* | *-* | *-* |
| BBS10 | -0.09 | (-0.63, 0.44) | 0.721 |
| Gender |  |  |  |
| Female | *Reference* | *-* | *-* |
| Male | 0.07 | (-0.40, 0.53) | 0.778 |
| BMI | 0 | (-0.03, 0.03) | 0.813 |
| Age | -0.02 | (-0.04, 0.00) | 0.121 |
| **Triglyceride (mmol/L)** | | | |
| Genotype |  |  |  |
| BBS1 | *Reference* | *-* | *-* |
| BBS10 | 0.45 | (-0.07,0.97) | 0.09 |
| Gender |  |  |  |
| Female | *Reference* | *-* | *-* |
| Male | 0.53 | (0.08, 0.99) | **0.023** |
| BMI | 0.02 | (-0.01, 0.05) | 0.158 |
| Age | -0.01 | (-0.03,0.01) | 0.477 |
| **LDL cholesterol (mmol/L)** | | | |
| Genotype |  |  |  |
| BBS1 | *Reference* | *-* | *-* |
| BBS10 | -0.25 | (-0.74, 0.24) | 0.312 |
| Gender |  |  |  |
| Female | *Reference* | *-* | *-* |
| Male | -0.18 | (-0.61, 0.25) | 0.403 |
| BMI | 0 | (-0.03, 0.02) | 0.775 |
| Age | -0.02 | (-0.04, 0.00) | 0.064 |
|  |  |  |  |
| **HDL cholesterol (mmol/L)** | | | |
| Genotype |  |  |  |
| BBS1 | *Reference* | *-* | *-* |
| BBS10 | -0.06 | (-0.22, 0.11) | 0.506 |
| Gender |  |  |  |
| Female | *Reference* | *-* | *-* |
| Male | 0 | (-0.15, 0.14) | 0.95 |
| BMI | 0 | (-0.01, 0.01) | 0.7 |
| Age | 0.01 | (-0.00, 0.01) | 0.09 |
| **Urea (mmol/L)** | | | |
| Genotype |  |  |  |
| BBS1 | *Reference* | *-* | *-* |
| BBS10 | 1.42 | (-1.33, 4.16) | 0.3 |
| Age | 0.06 | (-0.01, 0.13) | 0.107 |
| **Creatinine (umol/L)** | | | |
| Genotype |  |  |  |
| BBS1 | *Reference* | *-* | *-* |
| BBS10 | 30.44 | (-7.41, 68.29) | 0.113 |
| Age | 0.63 | (-0.64,1.90) | 0.322 |
| Gender |  |  |  |
| Female | *Reference* | *-* | *-* |
| Male | -3.87 | (-38.18, 30.44) | 0.822 |
| **Alb/Cr ratio** | | | |
| Genotype |  |  |  |
| BBS1 | *Reference* | *-* | *-* |
| BBS10 | -3.63 | (-20.66, 13.40) | 0.668 |
| Age | 0 | (-0.46, 0.47) | 0.996 |
| Gender |  |  |  |
| Female | *Reference* | *-* | *-* |
| Male | -5.99 | (-20.18, 8.19) | 0.397 |
| **Gamma Glutamyl Transferase (U/L)** | | | |
| Genotype |  |  |  |
| BBS1 | *Reference* | *-* | *-* |
| BBS10 | 20.08 | (-3.80, 43.97) | 0.095 |
| Gender |  |  |  |
| Female | *Reference* | *-* | *-* |
| Male | **18.3** | **(1.00, 35.61)** | **0.039** |
| BMI | **1.18** | **(0.05, 2.31)** | **0.041** |
| Age | 0.3 | (-0.35, 0.95) | 0.352 |

*p-value obtained from linear regression model
