# Supplementary material for: Genetic predictors of cardiovascular morbidity in Bardet–Biedl syndrome
Source: Clin Genet. 2014 Apr 8;87(4):343–9. doi: 10.1111/cge.12373 (PMC4402025; doi:10.1111/cge.12373)
Supplement: Supplementary file 4 — Table S4. Mutation type-phenotype comparison: multivariable comparison of selected parameters associated with cardiovascular disease. Homozygous missense, heterozygous truncating and missense and homozygous truncating. Statistically significant results are highlighted in bold. [file cge0087-0343-sd4.doc]

**Table 4**

|  | **β Estimate** | **95.0% CI** | **p-value*** |
| --- | --- | --- | --- |
| **Systolic blood pressure (mmHg)** | | | |
| Mutation type |  |  |  |
| Missense/missense | *Reference* | *-* | *-* |
| Missense/null | -6.26 | (-16.58, 4.06) | 0.229 |
| Null/null | 2.2 | (-6.40, 10.79) | 0.61 |
| Age | 0.21 | (-0.10, 0.51) | 0.183 |
| Height (cm) | 0.1 | (-0.10, 0.30) | 0.32 |
| Gender |  |  |  |
| Female | *Reference* | *-* | *-* |
| Male | 3.62 | (-3.39, 10.62) | 0.305 |
| BMI | 0.33 | (-0.16, 0.81) | 0.184 |
| **Diastolic blood pressure (mmHg)** | | | |
| Mutation type |  |  |  |
| Missense/missense | *Reference* | *-* | *-* |
| Missense/null | -4.14 | (-13.14, 4.86) | 0.36 |
| Null/null | 1.89 | (-5.67, 9.45) | 0.616 |
| Age | 0.26 | (-0.02, 0.53) | 0.064 |
| Height (cm) | -0.02 | (-0.22, 0.18) | 0.865 |
| Gender |  |  |  |
| Female | *Reference* | *-* | *-* |
| Male | 2.4 | (-3.86, 8.67) | 0.444 |
| BMI | 0.05 | (-0.37, 0.47) | 0.815 |
| **White cell count (10^9^L)** | | | |
| Mutation type |  |  |  |
| Missense/missense | *Reference* | *-* | *-* |
| Missense/null | 1.16 | (-0.51, 2.83) | 0.169 |
| Null/null | 0.58 | (-0.73, 1.90) | 0.377 |
| Age | -0.01 | (-0.05, 0.03) | 0.709 |
| **CRP (mg/L)** | | | |
| Mutation type |  |  |  |
| Missense/missense | *Reference* | *-* | *-* |
| Missense/null | -0.65 | (-4.42, 3.12) | 0.729 |
| Null/null | **5.33** | **(1.99, 8.68)** | **0.002** |
| Age | 0.06 | (-0.05, 0.17) | 0.272 |
| BMI | 0.14 | (-0.03, 0.31) | 0.11 |
| **C peptide (ng/ml)** | | | |
| Mutation type |  |  |  |
| Missense/missense | *Reference* | *-* | *-* |
| Missense/null | -433.78 | (-1673.12, 805.55) | 0.479 |
| Null/null | 635.54 | (-259.46, 1530.55) | 0.157 |
| BMI | 27.86 | (-31.80, 87.53) | 0.347 |
| Blood glucose | 94.24 | (-76.73, 265.20) | 0.268 |
| **Cholesterol (mmol/L)** | | | |
| Mutation type |  |  |  |
| Missense/missense | *Reference* | *-* | *-* |
| Missense/null | -0.34 | (-1.03, 0.35) | 0.326 |
| Null/null | -0.07 | (-0.63, 0.50) | 0.814 |
| Gender |  |  |  |
| Female | *Reference* | *-* | *-* |
| Male | 0.09 | (-0.38, 0.56) | 0.699 |
| BMI | 0 | (-0.03, 0.03) | 0.88 |
| Age | -0.02 | (-0.04, 0.00) | 0.118 |
| **Triglycerides (mmol/L)** | | | |
| Mutation type |  |  |  |
| Missense/missense | *Reference* | *-* | *-* |
| Missense/null | 0 | (-0.67, 0.67) | 0.996 |
| Null/null | **0.56** | **(0.01, 1.11)** | **0.048** |
| Gender |  |  |  |
| Female | *Reference* | *-* | *-* |
| Male | **0.52** | **(0.07, 0.98)** | **0.026** |
| BMI | **0.03** | **(0.00, 0.06)** | **0.05** |
| Age | -0.01 | (-0.03, 0.01) | 0.452 |
| **LDL cholesterol (mmol/L)** | | | |
| Mutation type |  |  |  |
| Missense/missense | *Reference* | *-* | *-* |
| Missense/null | -0.18 | (-0.82, 0.47) | 0.582 |
| Null/null | -0.17 | (-0.69, 0.36) | 0.531 |
| Gender |  |  |  |
| Female | *Reference* | *-* | *-* |
| Male | -0.16 | (-0.59, 0.28) | 0.474 |
| BMI | -0.01 | (-0.04, 0.02) | 0.561 |
| Age | -0.02 | (-0.03,0.00) | 0.089 |
| **HDL cholesterol (mmol/L)** | | | |
| Mutation type |  |  |  |
| Missense/missense | *Reference* | *-* | *-* |
| Missense/null | -0.17 | (-0.37, 0.04) | 0.112 |
| Null/null | -0.16 | (-0.33, 0.01) | 0.073 |
| Gender |  |  |  |
| Female | *Reference* | *-* | *-* |
| Male | 0 | (-0.14, 0.14) | 0.974 |
| BMI | 0 | (-0.01, 0.01) | 0.546 |
| Age | 0 | (-0.00, 0.01) | 0.166 |
| **Urea (mmol/L)** | | | |
| Mutation type |  |  |  |
| Missense/missense | *Reference* | *-* | *-* |
| Missense/null | 3.16 | (-0.16, 6.48) | 0.062 |
| Null/null | 1.18 | (-1.91, 4.28) | 0.439 |
| Age | 0.06 | (-0.02, 0.13) | 0.125 |
| **Creatinine (umol/L)** | | | |
| Mutation type |  |  |  |
| Missense/missense | *Reference* | *-* | *-* |
| Missense/null | 20.29 | (-30.19, 70.76) | 0.424 |
| Null/null | 33.53 | (-7.93, 74.99) | 0.111 |
| Age | 0.7 | (-0.60, 1.99) | 0.286 |
| Gender |  |  |  |
| Female | *Reference* | *-* | *-* |
| Male | -7.21 | (-41.52, 27.09) | 0.675 |
| **Albumin/creatinine ratio** | | | |
| Mutation type |  |  |  |
| Missense/missense | *Reference* | *-* | *-* |
| Missense/null | 19.63 | (-1.08, 40.35) | 0.062 |
| Null/null | 1.07 | (-15.90, 18.04) | 0.899 |
| Age | 0.09 | (-0.38, 0.56) | 0.703 |
| Gender |  |  |  |
| Female | *Reference* | *-* | *-* |
| Male | -4.18 | (-17.75, 9.40) | 0.535 |
| **Gamma Glutamyl Transferase (U/L)** | | | |
| Mutation type |  |  |  |
| Missense/missense | *Reference* | *-* | *-* |
| Missense/null | **44.22** | **(17.90, 70.54)** | **0.002** |
| Null/null | **29.32** | **(8.72, 49.91)** | **0.007** |
| Gender |  |  |  |
| Female | *Reference* | *-* | *-* |
| Male | **17.94** | **(4.12, 31.76)** | **0.013** |
| BMI | **1.05** | **(0.15, ,1.95)** | **0.025** |
| Age | 0.24 | (-0.28, 0.76) | 0.349 |

*p-value obtained from linear regression model
